# Shape Information in Repeated Glucose Curves during Pregnancy Provided Significant Physiological Information for Neonatal Outcomes

**DOI:** 10.1371/journal.pone.0090798

**Published:** 2014-03-11

**Authors:** Kathrine Frey Frøslie, Jo Røislien, Elisabeth Qvigstad, Kristin Godang, Jens Bollerslev, Tore Henriksen, Marit B. Veierød

**Affiliations:** 1 Department of Biostatistics, Institute of Basic Medical Sciences, University of Oslo, Oslo, Norway; 2 Norwegian Resource Centre for Women's Health, Division of Obstetrics and Gynaecology, Oslo University Hospital Rikshospitalet, Oslo, Norway; 3 Section of Specialised Endocrinology, Department of Medicine, Oslo University Hospital, Oslo, Norway; 4 Faculty of Clinical Medicine, University of Oslo, Oslo, Norway; 5 Division of Obstetrics and Gynaecology, Oslo University Hospital Rikshospitalet, Oslo, Norway; University of Barcelona, Faculty of Biology, Spain

## Abstract

**Objective:**

To use multilevel functional principal component analysis to exploit the information inherent in the shape of longitudinally sampled glucose curves during pregnancy, and to analyse the impact of glucose curve characteristics on neonatal birth weight, percentage fat and cord blood C-peptide.

**Study Design and Setting:**

A cohort study of healthy, pregnant women (n = 884). They underwent two oral glucose tolerance tests (gestational weeks 14–16 and 30–32), which gave two glucose curves per woman.

**Results:**

Glucose values were higher, and peaked later in third trimester than in early pregnancy. The curve characteristic “general glucose level” accounted for 91% of the variation across visits, and 72% within visits. The curve characteristics “timing of postprandial peak”, and “oscillating glucose levels” accounted for a larger part of the variation within visits (15% and 8%), than across visits (7% and <2%). A late postprandial peak during pregnancy, and high general glucose levels in third trimester had significant, positive effects on birth weight (p<0.05). Generally high glucose levels during pregnancy had a significant, positive impact on neonatal percentage fat (p = 0.04). High general glucose level in third trimester had a significant, positive impact on cord blood C-peptide (p = 0.004).

**Conclusion:**

Shape information in entire OGTT curves provides significant physiological information of importance for several outcomes, and may contribute to the understanding of the metabolic changes during pregnancy.

## Introduction

High maternal glucose levels in pregnancy have adverse short-term and long-term health effects for both the mother and the child [1]–[5]. The Hyperglycemia and Adverse Pregnancy Outcomes (HAPO) study investigated glucose intolerance less severe than that in overt diabetes mellitus, and demonstrated effects on the risk of adverse pregnancy outcomes [1]: Positive, linear effects were found for the fasting, one-hour (1-h) and two-hour (2-h) values from oral glucose tolerance tests (OGTTs). Other studies reporting associations between high maternal glucose levels and adverse pregnancy outcomes have used a variety of simple glucose measures, e.g. the fasting value, the 2-h value, area under the curve (AUC), impaired fasting glucose, gestational diabetes (GDM) diagnosis or HbA1c [6]–[12].

Changes in glucose metabolism during pregnancy include increasing insulin resistance and increasing gluconeogenesis in the liver [13]. Counterintuitive to this, longitudinal studies have reported a decrease in fasting glucose levels during pregnancy, particularly during the first trimester [13]–[15]. However, concomitantly with the decrease in fasting glucose, elevated postprandial levels during pregnancy have been reported [9], [16], [17]. Some studies have described glucose curves or glucose data at different gestational ages and longitudinal changes in these curves and data during pregnancy [9], [16], [18], [19]. Few studies have analysed the impact of information in the shape of entire OGTT glucose curves [20], and except from one study [16],we are not aware of statistical analysis of longitudinal change in glucose curves during pregnancy. Also, few have studied the impact of change in glucose levels during pregnancy on neonatal outcomes [21], [22].

Functional data analysis (FDA) is a collection of statistical methods developed to analyse curve data [23], [24]. In FDA a set of temporal observations is treated as a single, functional object. The statistical analysis is based on this continuous function (curve), rather than on the original discrete data points. Information from the curve as a whole is extracted.

In a previous article [20], we used FDA to extract curve shape information from glucose curves from OGTT at one time point in pregnancy (gestational weeks 14–16), and compared it with the information from commonly used simple summary measures. We found that FDA extracted physiologically interpretable and clinically interesting characteristics of the glucose response, which was not identified by simple summary measures, and would otherwise be missed [20]. In particular, the curve shape characteristic “time to peak”, discriminated between women with and without gestational diabetes later in pregnancy, while the simple summary measures (including AUC) did not. Based on these findings, we now extend the analysis to study the shape inherent in glucose curves from two visits during pregnancy. The aim is to extract physiologically interpretable curve shape characteristics from longitudinally sampled glucose curves during pregnancy, and to incorporate such information in explanatory models. To our knowledge, this is the first study to use all information in longitudinally collected glucose curves, and to analyse the effect of such information on neonatal outcomes.

The STORK study, a Norwegian prospective cohort study of 1031 healthy, pregnant women, provided OGTT data from gestational weeks 14–16 and 30–32 [25]. Using FDA methodology developed by Di et al [26] and Crainiceanu and Goldsmith [27], we performed a multilevel FDA of the OGTT data, and extracted essential characteristics of the OGTT glucose curves from gestational weeks 14–16 and 30–32. We then studied the effect of these characteristics on the neonatal outcomes birth weight, percentage fat and C-peptide in cord blood.

## Methods

### Ethics Statement

The study was approved by the Regional Committee for Medical Research Ethics, Southern Norway, Oslo, Norway (reference number S-01191), and performed according to the Declaration of Helsinki. All participating women provided written informed consent.

### Participants and data

The STORK study is a prospective cohort of 1031 healthy, Norwegian women of Scandinavian heritage who registered for obstetric care at Oslo University Hospital Rikshospitalet from 2001 to 2008. The overall aim of the study was to extend insights into maternal metabolic syndrome and determinants of foetal macrosomia [28]. Exclusion criteria were multiple pregnancies, known pre-gestational diabetes, and severe chronic diseases (pulmonary, cardiac, gastrointestinal or renal). Gestational age at inclusion was based on the Naegele's rule, and gestational ages at the other visits and at birth were based on routine ultrasound at weeks 17–19. Age, parity, smoking habits, height, weight, fasting insulin and a 75 g OGTT were recorded at inclusion at weeks 14–16. Weight, fasting insulin and OGTT were also recorded at weeks 30–32.

Blood samples were drawn in the morning, between 0730 and 0830 after an overnight fast, and were obtained from veni-puncture in tubes containing Ethylenediaminetetraacetic acid (EDTA). Plasma glucose was measured immediately in a drop of fresh, whole EDTA blood. During the OGTT, blood samples were taken every 30 minute for 2 hours. Glucose measurements were done by the Accu-Chek Sensor (ACS) glucometer (Roche Diagnostics GmbH, Mannheim, Germany). Due to an unexpected increasing trend in the fasting glucose measurements over the 7 years of recruitment, all glucose measurements were de-trended prior to the analyses [29]. The mean yearly increase in the original fasting glucose values was 0.11 mmol/l, 95% CI (0.09, 0.12) mmol/l. After the de-trending, the mean yearly increase was 0.01 mmol/l (−0.003, 0.024) mmol/l. The umbilical cord blood was collected into EDTA tubes by the midwife, centrifuged for plasma separation and placed at −20°C for less than a month and stored long term at −80°C.

**Figure 1:**
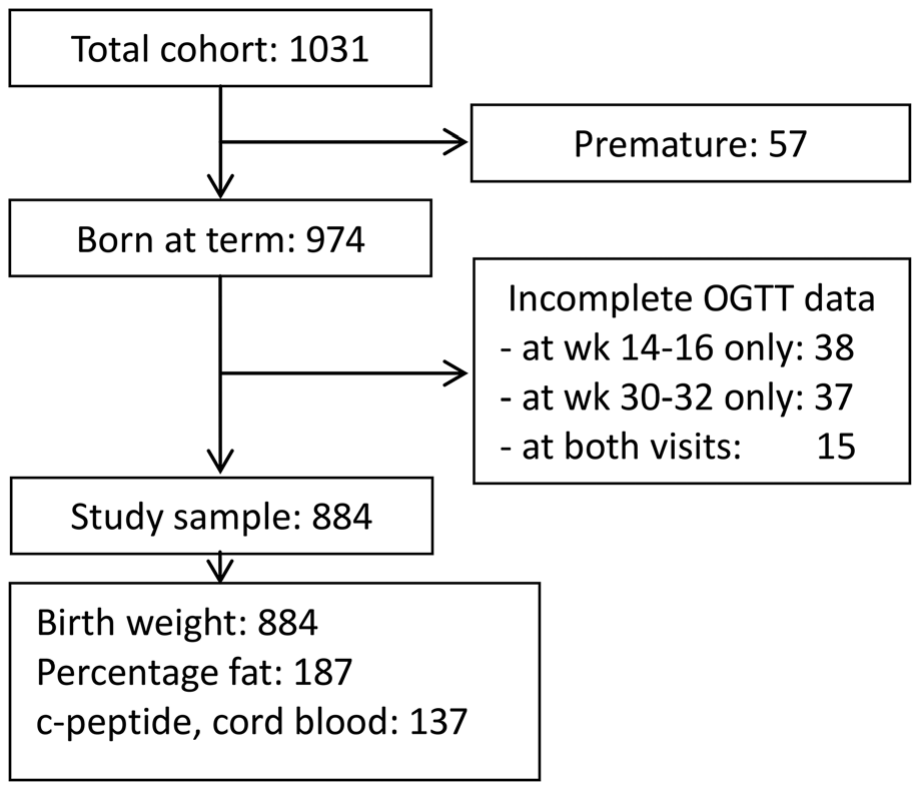
Flow chart.

Women with premature births or non-complete OGTT data were excluded, giving a study sample of 884 women and their neonates (Figure 1).

Birth weight was recorded within two hours after the birth. In a subsample of the cohort, the percentage of neonatal body fat was measured by Dual-energy X-ray absorptiometry (DXA) scanning, and C-peptide in cord blood from the time of birth was measured (Figure 1) [30], [31].

### Data description

Descriptive statistics of registered data are presented as mean, standard deviation (SD) and range, or frequency and percentage (%).

### Fitting continuous and individually smoothed curves

The OGTT measurements from the two visits were converted into 884 continuous, smooth OGTT glucose curves (hereafter called glucose curves), from gestational weeks 14–16, and 884 continuous, smooth glucose curves from gestational weeks 30–32. The curve fitting procedure was based on B-splines basis functions and is described in Appendix S1.

### The functional multilevel model

When functional data like glucose curves are observed on two or more occasions for each individual, we apply a multilevel model for functional data to extract information [26], [27], as described below. See Appendix S1 for details.

**Figure 2:**
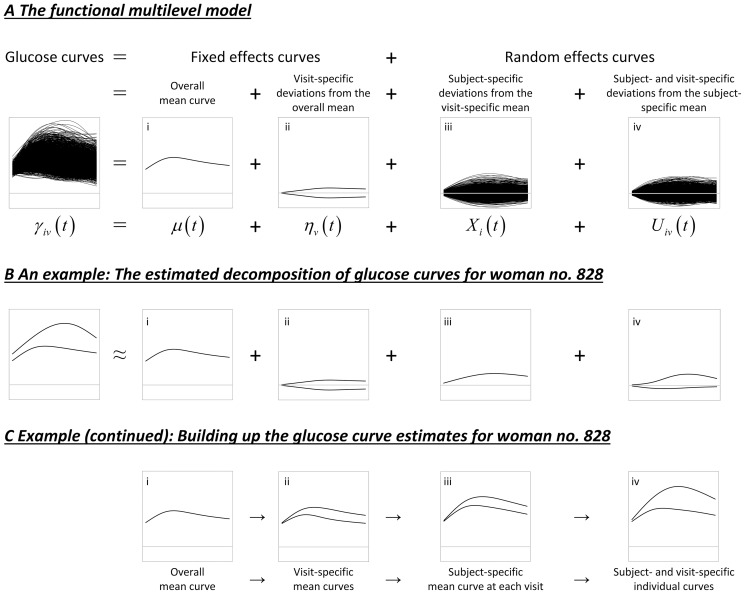
The functional multilevel model. In all plots in the figure, the horizontal axis is time during the 2−2 to 12.5 mmol/l. The horizontal, grey line is 0 mmol/l. 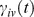 is the glucose curve from 0 to 120 min for woman 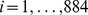 at visit 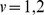; 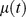 is the overall mean glucose curve; 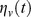 is the visit-specific deviation from the overall mean curve; 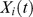 is the subject-specific deviation from the visit-specific mean curve; and 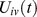 is the subject- and visit-specific deviation from the subject-specific mean curve.

Assume that the individual, true blood glucose curve 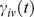 for woman 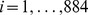 at visit 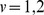 in the continuous time span from 0 to 120 minutes, 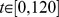, can be decomposed into fixed and random effects curves (Figure 2A), and expressed as a multilevel model of functional data
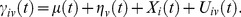
(1)Here the fixed effects curves are the overall mean glucose curve 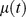 (Figure 2Ai), and the mean visit-specific deviation from the overall mean curve, 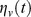 (Figure 2Aii). Together, these terms constitute the visit-specific mean curve, 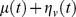 (Figure 2Cii). The random effects curves are 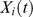, the subject-specific deviation from the visit-specific mean curve (Figure 2Aiii), and 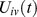, the subject- and visit-specific deviation from the subject-specific mean curve (Figure 2Aiv).

### Extracting common temporal characteristics: functional principal component (FPC) curves

An important task in FDA is to quantify the common characteristics of a set of curves. The common characteristics of the curves 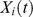, 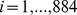 and of the curves 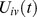, 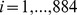 and 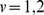 in expression (1) (Figure 2Aiii and 2Aiv) are found by functional principal component analysis (FPCA). FPCA extracts FPC curves that describe characteristics associated with the largest proportions of the variation in the curves. See Appendix S1 for details. The FPC curves estimated in the multilevel FPCA may be interpreted and labelled according to the physiological information they exhibit.

### Estimating FPC scores

In order to quantify each individual glucose curve's characteristics, we estimate individual scores for each FPC curve. A woman's FPC scores reflect how her individual curve trajectories at the two visits correspond to the common characteristics expressed by the FPC curves. Then we can study how glucose curve characteristics vary from woman to woman, and from visit to visit.

A woman's scores for the FPC curves of 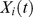 quantify her subject-specific deviation from the visit-specific mean curve, i.e. the important characteristics of her glucose curves *across* visits (Figure 2Biii and 2Ciii). Her scores for the FPC curves of 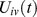 quantify her subject- and visit-specific deviation from her subject-specific mean curve, i.e. the characteristics of the residual variation *within* a visit (Figure 2Biv and 2Civ).

By combining equation (1) with the FPC curves and corresponding estimated FPC scores, an individual glucose curve can be expressed as the sum of the visit-specific mean, 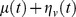
_,_ and a linear combination of a small number of the FPC curves for 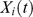 and 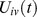 (Equation (3) in Appendix S1) [26], [27].

FPC curves are often illustrated by plots showing how an individual curve differs from the mean curve if the FPC scores are high or low, rather than plots of the FPC curves directly.

### Correlation between FPC scores and traditional AUC

The traditional, simple summary measure AUC was calculated directly from the de-trended glucose measurements by the trapezoid method. Pearson's correlation coefficients (*r*) were used to compare AUC with FPC scores.

### Functional information in regression analyses

The impact of glucose curve characteristics on the neonatal outcomes birth weight, percentage fat and C-peptide in cord blood were estimated using linear regression with FPC scores from the multilevel FPCA as explanatory variables. The interpretation of the effect estimates is based on the physiological interpretation of the FPC scores. Adjusted effect estimates were found by multiple linear regression analyses with most FPC scores (the first subject- and visit-specific score at gestational weeks 14–16 was left out due to colinearity issues), early pregnancy BMI, age and parity as explanatory variables. The multivariable analyses involved stepwise variable selection procedures based on Akaike's information criterion (AIC), analyses of several models considered to be of importance, and considerations of physiological importance of the findings. Model diagnostics were thoroughly checked during the analysis. The final multiple models presented in the results section include only the variables identified by these procedures.

In supplementary analyses, we repeated the analyses of birth weight for the reduced samples where percentage fat and C-peptide were available.

### Software

All analyses were performed in R 3.0.0. The estimation of FPC scores was done by the R2WinBUGS package that runs WinBUGS from R [32]. The technical details are given in Appendix S1, and the implementation is given in the program code, which is available in Appendix S2.

## Results

### Data description

Characteristics of the study sample at inclusion at gestational weeks 14–16, at gestational weeks 30–32 and at birth are shown in Table 1. Except from a significantly lower proportion of smokers in the study sample (p = 0.01), no significant differences were found between the women and the neonates in the study sample, and those who were excluded (0.41≤*p*≤1.00). There was a small increase in fasting glucose and a large increase in fasting insulin from inclusion to weeks 30–32. The 2-h glucose levels were elevated in third trimester, and the prevalence of GDM increased from 0.3% at inclusion to 6% in third trimester.

**Table 1:** Sample characteristics.

		Study sample, n = 884*		Excluded, n = 90*
			Range	
	Maternal age (years)	31 (4)	19–42	31 (4)
	Para 0	461 (52%)		43 (48%)
	Daily smoker†	15 (2%)		6 (7%)
	Height (cm)	169 (6)	150–184	169 (6)
Inclusion	Gestational weeks	15.8 (1.3)	12.1–22.0	15.8 (1.4)
	Weight (kg)	69.8 (12.1)	44.6–123.1	70.3 (11.4)
	Body mass index (kg/m^2^)	24.5 (4.0)	17.2–44.0	24.4 (3.5)
	Fasting blood glucose (mmol/l)	4.0 (0.4)	2.6–5.3	
	120 min blood glucose (mmol/l)	4.1 (1.1)	1.2–7.8	
	Fasting insulin (pmol/l) median (Q_1_,Q_3_)	27 (18, 39)	8–305	
	GDM‡	3 (0.3%)		
Third trimester	Gestational week	31.2 (1.0)	26.0–35.4	31.2 (0.8)
	Weight gain from inclusion	7.7 (2.6)	−2–22	7.8 (2.1)
	Fasting blood glucose (mmol/l)	4.1 (0.5)	3.0–6.2	
	120 min blood glucose (mmol/l)	5.5 (1.3)	1.9–10.3	
	Fasting insulin (pmol/l) median (Q_1_,Q_3_)	41 (26, 61)	8–989	
	GDM‡	50 (6%)		
Birth	Gestational week	40.2 (1.2)	37.0–43.1	40.1 (1.2)
	Birth weight child (g)	3654 (481)	2315–5420	3697 (527)
	Total % fat§ (n = 187)	13.6 (2.4)	8–20	13.2 (2.1)
	C-peptide in cord blood (ng/ml) (n = 137)	1.1 (0.7)	0.1–5.0	1.1 (0.9)

Characteristics of the study sample and those excluded due to incomplete OGTT data. Results are presented as means (SDs) for continuous variables and frequencies (%) for categorical variables, unless otherwise stated.

*n may vary due to missing values.

† More than 1 cigarette/day.

‡ GDM; gestational diabetes: 120 min glucose at or above 7.8 mmol/l.

§ Percentage fat estimated by DXA scan.

### Fitted curves

**Figure 3:**
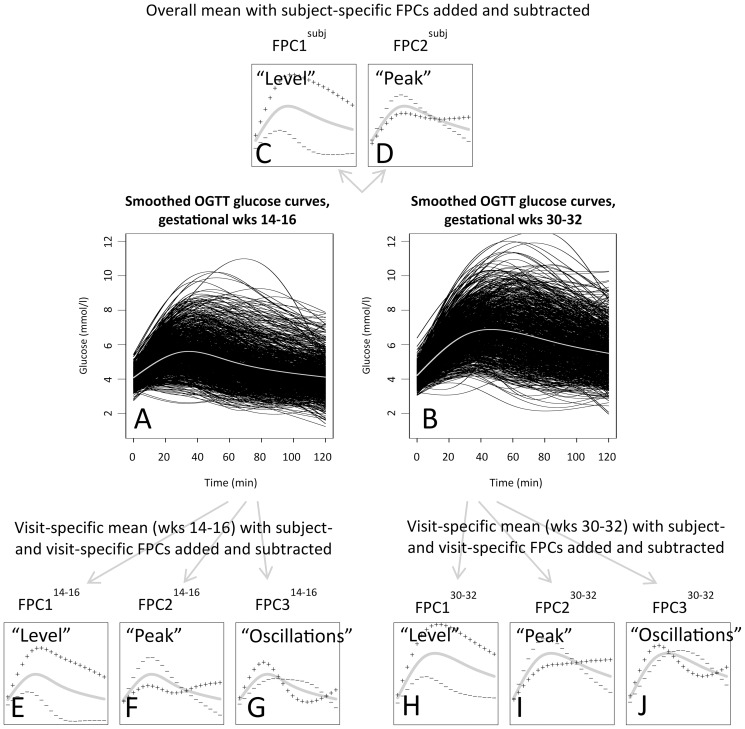
Smoothed glucose curves and results from the multilevel FPCA. Plots A and B show individually smoothed curves from gestational weeks 14–16 and 30–32 (black lines) and the visit-specific mean curves (grey lines). Plots C and D show the overall mean of the fitted curves (grey lines) and how the shape of an individual curve differs from the overall mean if a multiplum of the subject-specific FPC curves is added to (+) or subtracted from (−) the mean curve. Plots E–G and H–J show the visit-specific means at gestational weeks 14–16 and 30–32, respectively (grey lines), and how the shape of an individual curve differs from the visit-specific mean if a multiplum of the subject- and visit-specific FPC curves is added to (+) or subtracted from (−) this mean. The multiplums correspond to 2 SDs of the corresponding FPC scores.

The smoothed glucose curves at gestational weeks 14–16 (Figure 3A) and 30–32 (Figure 3B) showed large variations between the women at both visits. Glucose values were higher, and it took longer time for postprandial glucose levels to get back to fasting levels at gestational weeks 30–32 than at weeks 14–16.

### Common temporal characteristics: FPC curves and FPC scores

In the multilevel FPCA, the first two subject-specific FPCs explained 98% of the variation *across* visits, and the first three subject- and visit-specific FPCs explained 92% of the residual variation *within* visits. Further analyses were restricted to these FPC curves and the corresponding FPC scores (FPC1^subj^ and FPC2^subj^, and FPC1^14–16^, FPC2^14–16^, FPC3^14–16^, FPC1^30–32^, FPC2^30–32^ and FPC3^30–32^, respectively). The FPC1 and FPC2 curves had very similar temporal appearances across and within visits.

Figure 3C–J show how individual curves differ from the overall and visit-specific mean curves if the FPC^subj^, FPC^14–16^ and FPC^30–32^ scores are high or low. The dominating curve characteristic for the variation *across* visits, FPC1^subj^, accounting for 91% of this variation, was given the interpretation “general glucose level”. Women with high FPC1^subj^ scores had glucose curves above the overall mean, and a somewhat later postprandial peak, whereas women with low FPC1^subj^ scores had glucose curves below the overall mean (Figure 3C).

The second most important curve characteristic across visits (FPC2^subj^) was “timing of postprandial peak”. Women with low FPC2^subj^ scores had a clear early peak and low glucose values at the end of the OGTTs (Figure 3D). Women with high FPC2^subj^ scores had a later postprandial peak and high glucose values at the end of the OGTTs. This was seen in plots of individual curves from women with the lowest and highest FPC2^subj^ scores (plots not shown).

The dominating curve characteristic for the residual variation *within* visits (accounting for 72% of this variation), was “general glucose level within visits”, i.e. the general glucose level not accounted for by the general glucose level *across* visits. A woman with a high subject- and visit-specific FPC1^14–16^ (FPC1^30–32^) score had a glucose curve above the subject-specific mean at weeks 14–16 (30–32) (Figure 3E and 3H). An example of this is the upper curve in Figure 2Biv and 2Civ. Similarly, a woman with a low FPC1^14–16^ (FPC1^30–32^) score had a glucose curve below the subject-specific mean at weeks 14–16 (30–32). An example of this is the lower curve in Figure 2Biv and 2Civ.

The second most important curve characteristic for the variation within visits was “timing of postprandial peak within visits”. A woman with a low FPC2^14–16^ (FPC2^30–32^) score had a clear early peak, and low glucose values at the end of this OGTT (Figure 3F and 3I). A woman with a high FPC2^14–16^ (FPC2^30–32^) score at weeks 14–16 (30–32) had a later postprandial peak, with high glucose values at the end of this OGTT (plots of individual curves not shown).

The third most important curve characteristic for the variation within visits was “oscillating glucose within visits”. The glucose curves of women with high FPC3^14–16^ or FPC3^30–32^ scores had two postprandial peaks during the corresponding OGTT, whereas women with low FPC3^14–16^ or FPC3^30–32^ scores had only one glucose peak during the OGTT (Figure 3G and 3J). The characteristics “peak” and “oscillations” accounted for a smaller part of the variation *across* visits (7% and less than 2%), than *within* visits (15% and 8%).

**Figure 4:**
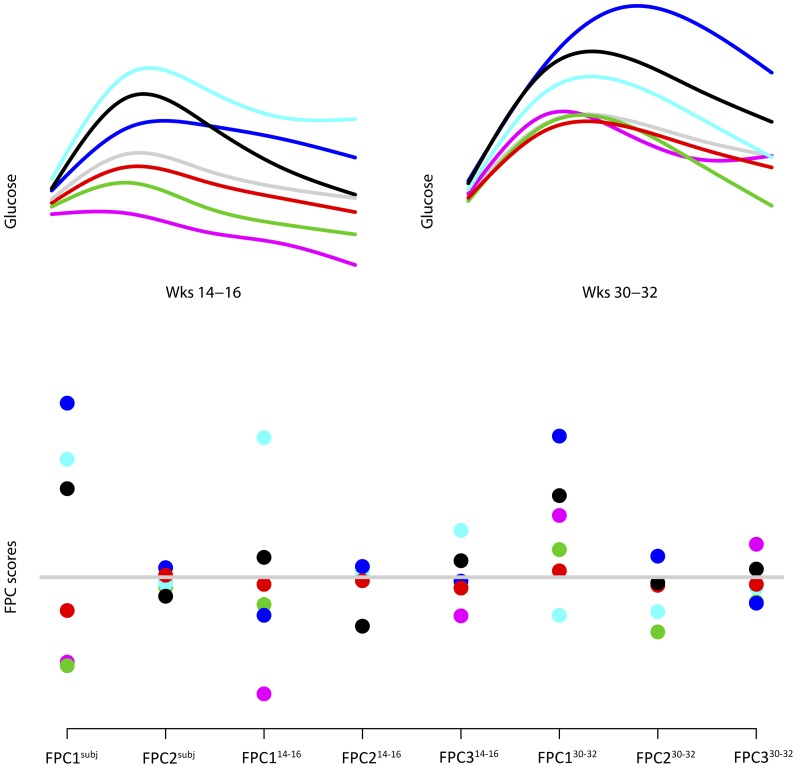
Examples of individual curves and corresponding scores. The upper, left plot shows the individual glucose curves from 6 women at gestational weeks 14–16, and the upper, right plot shows the glucose curves from the same 6 women at gestational weeks 30–32. The lower plot shows the FPC scores for the same 6 women. The grey curves in the upper plots are the mean glucose curves at gestational weeks 14–16 (left) and 30–32 (right). Correspondingly, the grey line in the lower plot is zero.

Figure 4 exemplifies the relation between individual glucose curves and corresponding FPC scores. The cyan, blue and black curves are glucose curves from three women in the study with curves above the mean (grey curve) at both visits. Consequently, the FPC1^subj^ scores were high. The women with the green, red and purple curves had low FPC1^subj^ scores. The woman with cyan curves and generally high glucose levels on both OGTTs, had a curve that was high above the mean at gestational weeks 14–16, but less so at gestational weeks 30–32. Thus, her FPC1^14–16^ score was high, and her FPC1^30–32^ score low.

There were strong correlations between FPC1^subj^ scores and AUC at weeks 14–16 and weeks 30–32 (*r* = 0.86 and 0.90, respectively), between FPC1^14–16^ and AUC^14–16^ (*r* = 0.73), and between FPC1^30–32^ and AUC^30–32^ (*r = *0.87). All correlations are given in Table S1. All FPC1 scores were positively correlated with BMI (0.12*≤r≤*0.35).

### Regression analyses

Crude analyses showed significant positive effects on the three neonatal outcomes of the general glucose level across visits (FPC1^subj^), and of the subject- and visit-specific general glucose level at weeks 30–32 (FPC1^30–32^) (Table 2). There was also a significant effect of a subject-specific late peak (FPC2^subj^) on birth weight.

**Table 2:** Regression analyses.

Outcome:		Birth weight, n = 868			Percentage fat of newborn, as measured by DXA, n = 185			C-peptide in cord blood, n = 134		
Crude estimates		B	95% CI	p	B	95% CI	p	B	95% CI	p
Subject-specific	FPC1^subj^, “Level”	51	(30,73)	<0.001	0.34	(0.11,0.57)	<0.001	0.08	(0.00,0.16)	0.05
	FPC2^subj^, “Peak”	−112	(−205,−18)	0.02	−1.10	(−2.26,0.06)	0.06	−0.11	(−0.53,0.31)	0.61
Wks 14–16	FPC1^14–16^, “Level”	−21	(−50,8)	0.16	−0.02	(−0.35,0.31)	0.91	−0.09	(−0.21,0.02)	0.11
	FPC2^14–16^, “Peak”	−23	(−94,47)	0.52	−0.37	(−1.12,0.38)	0.33	−0.12	(−0.39,0.15)	0.38
	FPC3^14–16^, “Oscill”	28	(−68,124)	0.56	0.43	(−0.64,1.49)	0.43	−0.30	(−0.69,0.08)	0.12
Wks 30–32	FPC1^30–32^, “Level”	51	(26,76)	<0.001	0.26	(0.00,0.52)	0.05	0.14	(0.05,0.23)	<0.001
	FPC2^30–32^, “Peak”	−29	(−87,28)	0.32	−0.16	(−0.89,0.56)	0.66	0.07	(−0.20,0.34)	0.60
	FPC3^30–32^, “Oscill”	−20	(−101,60)	0.62	−0.66	(−1.59,0.27)	0.16	−0.16	(−0.47,0.16)	0.32
Adjusted estimates from multivariable analyses*										
Subject-specific	FPC1^subj^, “Level”				0.25	(0.01,0.49)	0.04			
	FPC2^subj^, “Peak”	−92	(−181,−3)	0.04	−0.89	(−2.03,0.26)	0.13			
Wks 14–16	FPC1^14–16^, “Level”									
	FPC2^14–16^, “Peak”									
	FPC3^14–16^, “Oscill”							−0.34	(−0.72,0.03)	0.07
Wks 30–32	FPC1^30–32^, “Level”	38	(14,62)	0.002				0.13	(0.04,0.23)	0.004
	FPC2^30–32^, “Peak”									
	FPC3^30–32^, “Oscill”									
BMI	Wks 14–16	24	(16,32)	<0.001	0.09	(−0.01,0.18)	0.06	0.02	(−0.01,0.05)	0.13
Parity		189	(128,251)	<0.001						

Results from univariable and multivariable regression analyses, with birth weight, neonatal percentage fat, or C-peptide in cord blood as response variables.

*Multivariable analyses included all glucose variables except FPC1^14–16^, due to colinearity diagnostics. Variable selection was done by Akaike's information criterion.

In multivariable analyses of birth weight, only FPC2^sub^and FPC1^30–32^ scores remained significant (Table 2): Women with late postprandial peaks (low FPC2^subj^ scores) would be expected to have babies with higher birth weight than women with early postprandial peaks (high FPC2^subj^ scores), and women with the highest residual glucose levels in third trimester (high FPC1^30–32^ scores) would be expected to have babies with higher birth weight than women with low residual glucose levels in third trimester (low FPC1^30–32^ scores).

In multivariable analyses of neonatal percentage of fat, the effect of the FPC1^subj^ scores remained significant (Table 2): Women with generally high glucose levels during their OGTTs (high FPC1^subj^ scores) would be expected to have babies with a higher percentage of fat than women with generally low glucose levels during their OGTTs (low FPC1^subj^ scores). According to AIC, FPC2^subj^ scores also held information important for this outcome, although not statistically significant in the final model: Low FPC2^subj^ scores, implying late glucose peaks, corresponded with high values of neonatal percentage fat.

In multivariable analyses of C-peptide in cord blood, the effect of FPC1^30–32^ scores remained significant (Table 2): Women with high FPC1^30–32^ scores gave birth to babies with higher mean C-peptide than women with low FPC1^30–32^ scores. According to AIC, FPC3^14–16^ scores also held important information, although not statistically significant: The neonates of women with oscillating glucose curves (high FPC3^14–16^ scores) had somewhat lower C-peptide levels than those with one glucose peak during the OGTT (low FPC3^14–16^ scores).

Supplementary analyses showed that alternative models chosen due to physiological theories, and to explore the effects of colinearity, gave the same results for all three outcomes. Analyses of birth weight in reduced samples where percentage fat or C-peptide was available showed significant effects of FPC1^subj^ in both subsamples, of FPC2^14–16^ in the percentage fat subsample, and a non-significant contribution of FPC2^30–32^ in the C-peptide subsample.

## Discussion

The present study successfully used multilevel FDA to analyse changes in longitudinally observed glucose curves during pregnancy. The general glucose levels, in particular postprandial glucose, increased from early pregnancy to gestational weeks 30–32, and postprandial glucose peaked later in gestational weeks 30–32. The glucose characteristics extracted by FPCA had significant impact of glucose curve characteristics on birth weight, neonatal percentage of fat, and C-peptide in cord blood, demonstrating physiological relevance.

The physiological interpretation of FPC curves is essential for the usefulness of FPCA. The identification of the general glucose levels as the most dominant characteristics of individual glucose curves was supported by the strong associations between FPC1 scores and the AUCs. The elevated postprandial levels in third trimester, the small increase in fasting glucose, and the large increase in fasting insulin and prevalence of GDM from inclusion to weeks 30–32 (Table 1), are in accordance with an expected progressive insulin resistance among pregnant women [13]. This supports the finding of timing of postprandial peak as the second most important curve characteristic. The general glucose level accounted for a larger part of the variation across, than within visits, whereas the timing of the peak was more important for the variation within, than across visits. This is not surprising, as FPC2 represent more curvature than FPC1, and some of the curvature may be averaged out at the subject-specific level. The FPC3 curve was interpreted as “oscillating glucose within visits”, although the scarcely sampled glucose measurements at each OGTT could only reveal two glucose peaks. The term “oscillations” was chosen due to physiological theories [33], [34].

The regression results for birth weight in Table 2 were strengthened by the consistency of the results from alternative models. The different results for birth weight in the subsamples with percentage fat or cord blood C-peptide may be a consequence of colinearity issues in combination with the reduced sample size. Notably, in all these analyses, both a “general level” and a “timing of peak” characteristic were identified as important for birth weight.

The sample size in the present study was substantial, but the women were healthy and relatively homogenous. This may have caused less variation in the individual glucose curves and made it more difficult to extract important discriminating curve characteristics. Also, the scarce sampling of glucose during the OGTT is likely to obscure the extraction and interpretation of the curve characteristics. More physiologically interesting temporal details and better discriminating abilities of the FPCs may be expected in a more heterogeneous population, and from OGTT curves over more than 2 hours or with a more frequent OGTT sampling. With more measurements per OGTT, it is also possible to apply alternative smoothing strategies [27]. In this study, the smoothing involved both a roughness penalty when fitting individual curves, and leaving out the FPCs which explained the smallest part of the variation, i.e. those with the waviest appearance. This might have given a too conservative estimate of the amount of curvature in the individual curves, which again could have caused bias in the FPC scores and thereby affected the regression results. It is also possible that the methods of covariance matrix estimation did not perfectly separate the across and within variances, influencing the colinearity, and thereby the variable selection.

Compared to studies presenting intravenous glucose tolerance tests [35], the glucose measurements per woman per OGTT in our study were few. They were, nevertheless, samples from an underlying, continuous and temporal process, and this made FDA a natural choice of analysis [24]. Alternative analyses include ordinary principal component analysis (PCA) of the five glucose measurements, and using these PCA scores as input in the regression analyses instead of FPCA scores. With only five measurements per curve, and measurements taken at the same time points for each woman, this would be expected to extract similar information as the FDA. However, FDA emphasizes the basic assumption about continuity of the underlying process, provides interpretations of curve features in this context, and opens for analysis of the derivatives of the curves [24]. FDA is also easier to apply in situations with more frequent sampling, sampling at unequal time points, and missing data.

The finding of the general glucose level as the most important glucose curve characteristic is in accordance with the numerous studies focusing on elevated glucose of various types, e.g. fasting, 1-h, 2-h or HbA1c values, in diabetes research [1], [13], [36]. Also, the increase in postprandial values during pregnancy, and corresponding delay in postprandial peak, found in the present study, is supported by several earlier studies [9], [14], [16], [18], [37]. FPC1 scores were positively associated with BMI, indicating that higher BMI leads to generally higher glucose levels. This is also in accordance with physiological knowledge of obesity and insulin resistance [13], [38] and our recent findings [20]. Many studies have found a decline in fasting glucose during the first trimester of pregnancy [15], but an overview of longitudinal studies during pregnancy showed conflicting results concerning later pregnancy fasting glucose [15]. This justifies our findings of a small increase in fasting glucose from weeks 14–16, to 30–32. Hence, current knowledge of metabolic changes during pregnancy supports the interpretations of the FPCs as plausible and potentially important physiological characteristics.

The HAPO study is a reference study for the impact of maternal blood glucose levels on pregnancy outcomes, and has found statistically significant higher odds ratios for high birth weight, cord-blood serum C-peptide level and percentage body fat (above their respective 90^th^ percentiles), for high fasting, 1-hour and 2-hour glucose levels [1], [39]. This supports our findings of important impact of FPC1 scores, interpreted as “general glucose level”, on these outcomes. Other studies with a similar scope, but smaller sample sizes are also in accordance with these findings [2], [4], [6]–[8]. However, none of these studies addressed the impact of the dynamic regulation of the blood glucose, which is embedded in the FPC2 and FPC3 scores. Some studies have commented on the postprandial peak and birth outcomes [9], [22], [40], but to our knowledge, our previous study [20] is the only study that has formally investigated the impact of the timing of the postprandial peak.

We earlier found that for glucose curves from early pregnancy, the AUC was strongly correlated with FPC scores that provided information about the general glucose level during the OGTT, but not with scores providing information about timing of postprandial peak or oscillations, nor with the fasting or 2-h values [20]. This was also found in the present study (Table S1). Our recommendation is therefore to use AUC values rather than the fasting values or 2-h values, if FDA is not applied.

An important application of FDA techniques concerns research and clinical settings where continuous glucose monitoring devices are used [41]. Currently, many such studies restrict the analyses to simple summary measures like the mean glucose [17], [42], resulting in loss of potentially important information, as demonstrated in [20]. With the increased use of continuous glucose monitoring, there is a strong need for methods that can extract important information from curve data.

The HAPO study extended the Pedersen hypothesis about how maternal hyperglycemia affects the foetus [13] to the normal-glycaemic range, thereby giving rise to a comprehensive debate about the GDM diagnosis [11], [43]–[47]. In contrast to the WHO GDM criterion based on the fasting and 2-h value only [36], the new criteria suggested by the International Association of Diabetes and Pregnancy Study Group takes into consideration both the fasting, 1-h and 2-h OGTT values, with cut-off values based on risk estimates for adverse outcomes [47]. This implies that the new criteria indirectly address the dynamics in the curves. We have shown that modern statistical analysis can extract curve information reflecting glucose dynamics that is important for both maternal [20] and neonatal outcomes. This can contribute to a better understanding of the different stages in the development of unhealthy glucose metabolism, and to a more precise prediction of women at risk for maternal or foetal complications. Then, interventions targeted to modify glucose curves could be initiated before a GDM diagnosis is given, or treatment for it is necessary. Such interventions have been studied in pregnant and non-pregnant study samples [48]–[50]. Future studies should investigate whether such interventions also may affect pregnancy outcomes, and have positive long-term effects on maternal health.

In conclusion, the physiologically interpretable glucose curve characteristics extracted by FDA in the present analysis, and their statistically significant effects of on birth weight, neonatal percentage fat, and cord blood C-peptide, show that shape information inherent in entire glucose curves is important for several outcomes, and may contribute to the understanding of the metabolic changes during pregnancy. FDA techniques can also be used to capture important curve information from more frequently sampled glucose curves, such as the increasingly used continuous glucose monitoring devices.

## Supporting Information

Table S1 Correlations between glucose measurements and functional principal component scores.
(PDF)

Appendix S1 Multilevel functional data analysis (FDA), technical details.
(PDF)

Appendix S2 Program code.
(PDF)
