# Supplementary material for: Shape Information in Repeated Glucose Curves during Pregnancy Provided Significant Physiological Information for Neonatal Outcomes
Source: PLoS One. 2014 Mar 11;9(3):e90798. doi: 10.1371/journal.pone.0090798 (PMC3949679; doi:10.1371/journal.pone.0090798)
Supplement: Table S1 — Correlations between glucose measurements and functional principal component scores. (PDF) [file pone.0090798.s001.pdf]

Table S1

Correlations between glucose measurements and functional principal component scores in ***«Shape information in repeated glucose curves during pregnancy provided significant physiological information for neonatal outcomes»***, by Frøslie et al., PLOS ONE, 2014.

	OGTT, wks 14-16					OGTT, wks 30-32														
	Fasting	30 min	60 min	90 min	120 min	Fasting	30 min	60 min	90 min	120 min	AUC$^{14-16}$	AUC$^{30-32}$	FPC1^{subj} scores	FPC2^{subj} scores	FPC1$^{14-16}$ scores	FPC2$^{14-16}$ scores	FPC3$^{14-16}$ scores	FPC1$^{30-32}$ scores	FPC2$^{30-32}$ scores	FPC3$^{30-32}$ scores
OGTT, wks 14-16 Fasting	1.00																			
OGTT, wks 14-16 30 min	0.55	1.00																		
OGTT, wks 14-16 60 min	0.45	0.87	1.00																	
OGTT, wks 14-16 90 min	0.43	0.72	0.90	1.00																
OGTT, wks 14-16 120 min	0.42	0.58	0.71	0.83	1.00															
OGTT, wks 30-32 Fasting	0.47	0.34	0.32	0.31	0.28	1.00														
OGTT, wks 30-32 30 min	0.35	0.49	0.45	0.41	0.27	0.57	1.00													
OGTT, wks 30-32 60 min	0.33	0.52	0.54	0.51	0.37	0.49	0.85	1.00												
OGTT, wks 30-32 90 min	0.31	0.50	0.56	0.55	0.44	0.47	0.70	0.91	1.00											
OGTT, wks 30-32 120 min	0.27	0.38	0.45	0.50	0.48	0.44	0.50	0.65	0.81	1.00										
AUC$^{14-16}$	0.55	0.90	0.97	0.94	0.81	0.36	0.47	0.55	0.57	0.49	1.00									
AUC$^{30-32}$	0.36	0.53	0.56	0.54	0.42	0.58	0.87	0.97	0.95	0.78	0.58	1.00								
FPC1^{subj} scores	0.54	0.77	0.83	0.82	0.69	0.58	0.75	0.86	0.88	0.74	0.86	0.90	1.00							
FPC2^{subj} scores	-0.21	-0.29	-0.08	0.13	0.39	-0.19	-0.41	-0.17	0.07	0.45	-0.03	-0.09	-0.03	1.00						
FPC1$^{14-16}$ scores	0.24	0.61	0.72	0.70	0.66	-0.12	-0.15	-0.12	-0.09	-0.05	0.73	-0.12	0.29	0.09	1.00					
FPC2$^{14-16}$ scores	-0.08	-0.50	-0.32	0.02	0.30	0.01	-0.08	-0.13	-0.15	-0.15	-0.21	-0.14	-0.15	0.39	-0.09	1.00				
FPC3$^{14-16}$ scores	0.43	0.40	0.02	-0.07	0.31	0.18	0.12	0.05	0.03	0.05	0.16	0.07	0.13	-0.09	0.09	-0.08	1.00			
FPC1$^{30-32}$ scores	0.05	0.13	0.13	0.12	0.05	0.38	0.73	0.86	0.84	0.70	0.12	0.87	0.58	0.00	-0.54	-0.10	-0.04	1.00		
FPC2$^{30-32}$ scores	0.04	0.07	0.12	0.15	0.16	0.02	-0.35	-0.10	0.24	0.59	0.13	0.04	0.13	0.67	0.08	-0.30	-0.03	0.05	1.00	
FPC3$^{30-32}$ scores	0.10	-0.03	-0.10	-0.06	0.00	0.28	0.16	-0.24	-0.27	0.19	-0.06	-0.09	-0.07	0.07	-0.02	0.04	0.13	-0.11	0.06	1.00
