# Supplementary material for: Shape Information in Repeated Glucose Curves during Pregnancy Provided Significant Physiological Information for Neonatal Outcomes
Source: PLoS One. 2014 Mar 11;9(3):e90798. doi: 10.1371/journal.pone.0090798 (PMC3949679; doi:10.1371/journal.pone.0090798)
Supplement: Appendix S1 — Multilevel functional data analysis (FDA), technical details. (PDF) [file pone.0090798.s002.pdf]

Appendix S1: Multilevel functional data analysis (FDA)

The multilevel FDA was based on the works of Ramsay and Silverman [24], Di et al [26] and Crainiceanu and Goldsmith [27].

Fitting individually smoothed continuous curves

Let $\gamma_{iv}(t)$ be the underlying, true continuous and smooth glucose curve for woman $i=1,\ldots,N$ at visit $v=1,\ldots,V$. In our data, $N=884$, $V=2$ and $t\in[0,120]$. The estimation of individual curves $\hat{\gamma}_{iv}(t)$ from the observed discrete data points $y_{iv}(t_j)$, $j=1,\ldots,J$, is based on the measurement model $y_{iv}(t_j)=\gamma_{iv}(t_j)+\varepsilon_{ivj}$, where $\gamma_{iv}(t_j)$ is γ_{iv} evaluated at time t_j and $\varepsilon_{ivj}\sim\mathrm{N}(0,\sigma^2)$ is an error term. In our data, $J=5$. The individual curve estimates $\hat{\gamma}_{iv}(t)$ are found by subject-specific spline smoothing with B-splines basis functions and a roughness penalty [24].

The functional multilevel model

Assume that the individual continuous blood glucose curve $\gamma_{iv}(t)$ can be decomposed according to the model

$$\gamma_{iv}(t)=\mu(t)+\eta_v(t)+X_i(t)+U_{iv}(t). \qquad (1)$$

Here, $\mu(t)$ is the overall mean curve and $\eta_v(t)$ the mean visit-specific deviation from the overall mean curve, both assumed to be fixed effects curves. $X_i(t)$ and $U_{iv}(t)$ are assumed to be uncorrelated mean-zero stochastic processes, where $X_i(t)$ represent the subject-specific deviation from the visit-specific mean curve, $\mu(t)+\eta_v(t)$, and $U_{iv}(t)$ the subject- and visit-specific deviation from the subject-specific mean curve at visit *v*, $\mu(t)+\eta_v(t)+X_i(t)$.

Due to the large sample size, we assume that $\mu(t)$ can be estimated with negligible error by averaging the individual curve estimate $\hat{\gamma}_{iv}(t)$ over all subjects *i* and visits *v*,

$$\hat{\mu}(t)=\frac{1}{N\cdot V}\sum_{i,v}\hat{\gamma}_{iv}(t).$$

Similarly, we estimate the mean visit-specific deviation from the overall mean for visit v by

$$\hat{\eta}_v(t)=\left(\frac{1}{N}\sum_{i}\hat{\gamma}_{iv}(t)\right)-\hat{\mu}(t).$$

Extracting common temporal characteristics: functional principal component (FPC) curves

The common temporal characteristics for the stochastic processes $X_i(t)$ and $U_{iv}(t)$ can be found by functional principal component analysis (FPCA) of their temporal covariance surfaces K_X and K_U, for $t \in [0,120]$ and $s \in [0,120]$,

$$K_X(t,s) = \text{cov}(X_i(t), X_i(s))$$

and

$$K_U(t,s) = \text{cov}(U_{iv}(t), U_{iv}(s)).$$

To obtain estimates of K_X and K_U, we first subtract the estimated visit-specific mean curve, $\mu(t) + \eta_v(t)$, from (2), giving

$$W_{iv}(t) = X_i(t) + U_{iv}(t),$$

i.e. the combined subject-specific, and subject- and visit-specific stochastic process. Let $K_W(t,s)$ be the covariance surface of $W_{iv}(t)$. Since $X_i(t)$ and $U_{iv}(t)$ are assumed to be uncorrelated this is simply

$$K_W(t,s) = \text{cov}(W_{iv}(t), W_{iv}(s)) = K_X(t,s) + K_U(t,s).$$

Estimates of the covariance surfaces for $X_i(t)$ and $U_{iv}(t)$ can thus be based on $K_W(t,s)$. The only contribution to the between-visits covariance, $K_{W_{v \neq v'}}(t,s) = \text{cov}(W_{iv}(t), W_{iv'}(s))$, $v \neq v'$, is the subject-specific variation from $X_i(t)$, since $K_U(t,s) = \text{cov}(U_{iv}(t), U_{iv'}(s)) = 0$ for $v \neq v'$ when assuming $\text{cov}(X_i(t), U_{iv}(s)) = 0$. Hence, for $v \neq v'$,

$$K_{W_{v \neq v'}}(t,s) = \text{cov}(W_{iv}(t), W_{iv'}(s)) = \text{cov}(X_i(t), X_i(s)) = K_X(t,s).$$

That is, for $v \neq v'$, $K_X(t,s)$ can be estimated using a methods of moments estimator of $K_W(t,s)$. We denote this $\hat{K}_X(t,s)$. Consequently, for $v = v'$, $K_U(t,s)$ can be estimated by a corresponding methods of moments estimator

$$\hat{K}_U(t,s) = \hat{K}_{W_{v=v'}}(t,s) - \hat{K}_X(t,s).$$

Once $\hat{K}_X(t,s)$ and $\hat{K}_U(t,s)$ are available, the common temporal characteristics of $X_i(t)$ and $U_{iv}(t)$ can be estimated by principal component analysis of $\hat{K}_X(t,s)$ and $\hat{K}_U(t,s)$ as described in [26] and [27], extracting their corresponding functional principal component (FPC) curves $\psi_a^X(t)$, $a = 1,\ldots,A$ and $\psi_b^U(t)$, $b = 1,\ldots,B.$ The FPC curves $\psi_a^X(t)$, $a = 1,\ldots,A$ represent independent parts of the subject-specific temporal variability, and the FPC curves $\psi_b^U(t)$, $b = 1,\ldots,B$ represent independent parts of the subject- and visit-specific temporal variability.

Estimating FPC scores

The individual stochastic process $W_{iv}(t)$ can be expressed as linear combination of the FPC curves for $X_i(t)$ and $U_{iv}(t)$, by estimation of corresponding FPC scores. By retaining to the first A' principal component curves for $X_i(t)$, and the first B' principal component curves for $U_{iv}(t)$, the functional model for the joint stochastic process $W_{iv}(t)$ can be expressed as

$$W_{iv}(t) = \sum_{a=1}^{A'} \xi_{ia}\psi_a^X(t) + \sum_{b=1}^{B'} \zeta_{ivb}\psi_b^U(t) + \varepsilon_w(t); \qquad (2)$$

$$\xi_{ia} \sim N\left(0,\ \lambda_a^X\right);\ \zeta_{ivb} \sim N\left(0,\ \lambda_b^U\right);\ \varepsilon_w \sim N\left(0,\ \sigma_w^2\right),$$

where ξ_{ia} are the scores for $\psi_a^X(t)$, $a = 1,\ldots,A'$, ζ_{ivb} are the scores for $\psi_b^U(t)$, $b = 1,\ldots,B'$, λ_a^X is the eigenvalue of $\psi_a^X(t)$, λ_b^U is the eigenvalue of $\psi_b^U(t)$, and ε_w is an error term.

Implementation

The model in equation (3) can be implemented in WinBUGS to obtain estimates and corresponding estimation error, i.e. posterior distributions, of ξ_{ia} and ζ_{ivb} [27]. To completely specify the Bayesian model in WinBUGS, it is necessary to provide priors for all model parameters. Independent gamma priors with large dispersion were chosen as priors for all dispersion parameters in the model. The program code is available as supporting information SI1. The matrix notation needed for the implementation can be found in [26,27].

Individual curve estimates expressed by FPC curves and FPC scores

Combining equations (2) and (3) with estimated FPC scores $\hat{\xi}_{ia}$ and $\hat{\zeta}_{ivb}$, an individual glucose curve estimate $\tilde{\gamma}_{iv}(t)$ can be given as

$$\tilde{\gamma}_{iv}(t) = \hat{\mu}(t) + \hat{\eta}_v(t) + \sum_{a=1}^{A'} \hat{\xi}_{ia}\psi_a^X(t) + \sum_{b=1}^{B'} \hat{\zeta}_{ivb}\psi_b^U(t). \quad (3)$$

The glucose curve estimates $\hat{\gamma}_{iv}(t)$ from the penalised B-splines smoothing, and $\tilde{\gamma}_{iv}(t)$ from (4), will be similar, but not identical, as the linear combination in (4) is restricted to the first (A', B') FPC curves and scores. This restriction constitutes an additional smoothing of the observed glucose measurements.
