# Supplementary material for: Shape Information in Repeated Glucose Curves during Pregnancy Provided Significant Physiological Information for Neonatal Outcomes
Source: PLoS One. 2014 Mar 11;9(3):e90798. doi: 10.1371/journal.pone.0090798 (PMC3949679; doi:10.1371/journal.pone.0090798)
Supplement: Appendix S2 — Program code. (PDF) [file pone.0090798.s003.pdf]

Appendix S2: Program code for multilevel functional data analysis

```
###    Program code for the multilevel functional data analysis in «Shape information in repeated glucose curves during pregnancy provided
###    significant physiological information for neonatal outcomes», by Frøslie et al., PLOS ONE, 2014.
###    Parts of the program code are clsosely based on the code in «Bayesian Functional Data Analysis Using WinBUGS»,
###    by Crainiceanu and Goldsmith, Journal of Statistical Software, 2010.

        # Read spss data from the complete cohort of 1031 women

library(foreign)
spssdata                       <- read.spss("M:/Art5longitudinalFDA/STORK29.06.2011.sav",use.value.labels = TRUE, to.data.frame = TRUE)
spssdata                       <- spssdata[order(spssdata$id),]
spssdata$auc1                  <- 0.5*(spssdata$g01/2+spssdata$g11+spssdata$g21+spssdata$g31+spssdata$g41/2)
spssdata$auc3                  <- 0.5*(spssdata$g03/2+spssdata$g13+spssdata$g23+spssdata$g33+spssdata$g43/2)

        # Remove women with premature births or non-complete OGTT data to get the sudy sample of 884 women and their neonates

spssdata.term.complete.ogtt   <- spssdata[spssdata$gest>=37&is.na(spssdata$gest)==FALSE&
                                            is.na(spssdata$g01*spssdata$g11*spssdata$g21*spssdata$g31*spssdata$g41*
                                                  spssdata$g03*spssdata$g13*spssdata$g23*spssdata$g33*spssdata$g43)==FALSE,]

        # Make the 884x5-dimensional matrix of glucose data (raw data) from visit 1 and the one from visit 3

g1                             <- as.matrix(cbind(   spssdata.term.complete.ogtt$g01,spssdata.term.complete.ogtt$g11,

        spssdata.term.complete.ogtt$g21,spssdata.term.complete.ogtt$g31,spssdata.term.complete.ogtt$g41))
g3                             <- as.matrix(cbind(   spssdata.term.complete.ogtt$g03,spssdata.term.complete.ogtt$g13,

        spssdata.term.complete.ogtt$g23,spssdata.term.complete.ogtt$g33,spssdata.term.complete.ogtt$g43))

N_subj                         <- dim(g1)[1]         # 884
N_obs                          <- dim(g1)[2]         # 5
g                              <- rbind(g1,g3)       # dim: 1768,5
ng                             <- length(g[,1])

        # Plots. Remark: All plots in this code are not necessary, but may be useful for different purposes. The code is therefore kept in this file.
X11()
breaksuse                      <- c(0,30,60,90,120)
matplot(breaksuse,t(g[1:ng,]), type="l", lty=1, col="dark grey",lw=1,xlab="Time in minutes", ylab="Glucose in mmol/l")
X11()
par(mar=c(1,1,1,1))
matplot(breaksuse,t(g[1:ng,]), type="b", pch=20,lty=1,col="grey",lw=1,xaxt='n', yaxt='n',ann=FALSE,ylim=c(-0.5,12.5))
matplot(breaksuse,t(g[1:ng,]), col="black",pch=20,cex=2,add=TRUE)
abline(h=0,col="grey",lw=5)

        # Make B-spline basis. May have to install the fda package first: install.packages("fda")

library(fda)
mybasis                        <- create.bspline.basis(rangeval=c(0,120),norder=4, breaks=c(0,30,60,90,120))
```

```
        # Optimise lambda

#loglam                      <- seq(-30,20,0.05)
loglam                       <- seq(0,20,0.05)           # Example of alternativ range for loglam in case of local minimum (see below)
nlam                                 <- length(loglam)
dfsave                       <- rep(NA,nlam)
gcvsave                      <- rep(NA,nlam)

for (ilam in 1:nlam)
{
Lambda                       <- 10^loglam[ilam]
fdParobj                     <- fdPar(mybasis,2,lambda)
smoothlist                   <- smooth.basis(c(0,30,60,90,120),t(g),fdParobj)
dfsave[ilam]                 <- smoothlist$df
gcvsave[ilam]                <- sum(smoothlist$gcv)
}

        # Optimal value of lambda:

lambdaopt                    <- 10^loglam[gcvsave==min(gcvsave)]  #      lambdaopt <- 707.9458

        # Plots showing how gcv vary with loglambda.
        # (To ensute that the optimal value is not a local minimum. If so, choose an alternativ range for lambda (see above) )

X11()
par(mfrow=c(2,3))
plot(loglam,gcvsave,type="l")
plot(loglam,gcvsave,type="l",ylim=c(2200,4000),xlim=c(0,5))
plot(loglam,gcvsave,type="l",ylim=c(2300,2400),xlim=c(2.5,3.2))
abline(h=min(gcvsave),lty=2)
plot(loglam,gcvsave,type="l",ylim=c(2340,2360),xlim=c(2.7,3.1))
abline(h=min(gcvsave),lty=2)

        # Optimal smoothing of individual glucose curves, according to gcv criterion

fdParobj.opt                 <- fdPar(mybasis,2,lambdaopt)

        # Smoothed, individual curves (the basic units in further FDA), and corresponding evaluated function values

g1.smooth                    <- smooth.basis(c(0,30,60,90,120),t(g1),fdParobj.opt)      # These are the correct curves and glucose values
g3.smooth                    <- smooth.basis(c(0,30,60,90,120),t(g3),fdParobj.opt)      # for further analysis
eval.g1                      <- t(eval.fd(c(0,30,60,90,120),g1.smooth$fd))
eval.g3                      <- t(eval.fd(c(0,30,60,90,120),g3.smooth$fd))
eval.g                       <- rbind(eval.g1,eval.g3)
eval.error                   <- g-eval.g

        # Means of smoothed function values

overallmean.eval             <- colMeans(eval.g    ,na.rm=TRUE)
visitspecificmean1.eval      <- colMeans(eval.g1   ,na.rm=TRUE)
visitspecificmean3.eval      <- colMeans(eval.g3   ,na.rm=TRUE)
```

```
       # Minimal smoothing of mean curves (means of smoothed function values), to optain continuous mean curves

fdParobj.m                          <- fdPar(mybasis,2,1)

overallmean.eval.smooth             <- smooth.basis(c(0,30,60,90,120),overallmean.eval,fdParobj.m )
visitspecificmean1.eval.smooth      <- smooth.basis(c(0,30,60,90,120),visitspecificmean1.eval,fdParobj.m )
visitspecificmean3.eval.smooth      <- smooth.basis(c(0,30,60,90,120),visitspecificmean3.eval,fdParobj.m )


       # Plots
X11()
boxplot(eval.error,names=c("0","30","60","90","120"),ylim=c(-5.5,5.5),xaxt='n', yaxt='n',ann=FALSE)
X11()
par(mfrow=c(1,2))
plot(g1.smooth,lty=1,col="black",ylim=c(1,12),xlab="Time (min)",ylab="Glucose (mmol/l)",main="Smoothed OGTT glucose curves, \n gestational wks 14-16")
plot(visitspecificmean1.eval.smooth,lty=1,col="grey",lw=2,add=TRUE)
plot(g3.smooth,lty=1,col="black",ylim=c(1,12),xlab="Time (min)",ylab="Glucose (mmol/l)",main="Smoothed OGTT glucose curves, \n gestational wks 30-32")
plot(visitspecificmean3.eval.smooth,lty=1,col="grey",lw=2,add=TRUE)

       # Next step: Center the estimated visit 1 and 3 functional values on the visit-specific mean

g1.demeaned                         <- eval.g1 - matrix(rep(colMeans(eval.g1,na.rm=TRUE),nrow(eval.g1)),nrow=nrow(eval.g1),byrow=TRUE)
g3.demeaned                         <- eval.g3 - matrix(rep(colMeans(eval.g3,na.rm=TRUE),nrow(eval.g3)),nrow=nrow(eval.g3),byrow=TRUE)

       # Combine the de-meaned values in one matrix

g1and3.demeaned                     <- cbind(g1.demeaned,g3.demeaned)

       # Calculate the covariance matrix of the data centered at the visit-specific means.

big_covariance_v1and3               <- cov(g1and3.demeaned,use="pairwise.complete.obs")
round(big_covariance_v1and3,2)
big_correlation_v1and3              <- cor(g1and3.demeaned,use="pairwise.complete.obs")
round(big_correlation_v1and3,2)

       # Multilevel analysis        Gt: Gtotal     Gb: Gbetween   Gw: Gwithin
N                           <- N_obs
Gt                          <- (big_covariance_v1and3[1:N ,1: N]+big_covariance_v1and3[(N+1): (2*N),(N+1): (2*N)])/2
Gb                          <- (big_covariance_v1and3[1:N ,(N+1): (2*N)]+big_covariance_v1and3[(N+1): (2*N),1: N])/2 # This is the estimated Kx
Gw                          <- Gt-Gb                                                                                  # This is the estimated Ku

       # Remark: No covariances need here to be smoothed (as compared to the works of Crainiceanu and Di),
       # as we have smoothed the curves as our data preparation step.


       # Plots of the estimated Ku and Kx surfaces
X11()
par(mfrow=c(1,2))
contour(seq(0,120,30),seq(0,120,30),matrix(Gw,nrow=5,byrow=TRUE),main="Gwds (Original Gt matrix with Gb subtr from all elements.)")
contour(seq(0,120,30),seq(0,120,30),matrix(Gb,nrow=5,byrow=TRUE)   ,main="Gb (Original Gb matrix with Kx)")
```

```
       # PCA of the subject-specific level (Gb: Gbetween) and of the subject- and visit-specific level (Gw: Gwithin):

eigen(Gb)
eigen(Gw)

       # Decide the number of components that are kept at level 1 and 2. A general rule is to stop at the component where
       # the cumulative percentage of variance explained is greater than 90% and the variance explained by any single component
       # after is less than 1/N. The number of components are also no less than the pre-determined minimum values for K1 (1) or K2 (1).

Gbpst                                <- eigen(Gb)$values/sum(eigen(Gb)$values[1:4])
Gwpst                                <- eigen(Gw)$values/sum(eigen(Gw)$values)
K1                                   <- max( which(cumsum(Gbpst) < 0.9 | Gbpst > 1/N ) + 1, 1 ) # K1 = 2
K2                                   <- max( which(cumsum(Gwpst) < 0.9 | Gwpst > 1/N ) + 1, 1 ) # K2 = 3

       # Obtain the restricted number of level 1 and 2 eigenfunctions for Gw and  Gb (some are flipped due to the physiological interpretation)

dim.space_b                          <- 2   # level 1 (subject-specific)
psi_1                                <- cbind(-eigen(Gb)$vectors[,1],-eigen(Gb)$vectors[,2])

dim.space_w                          <-  3  # level 2 (subject/visit-specific)
psi_2                                <- cbind(-eigen(Gw)$vectors[,1],-eigen(Gw)$vectors[,2],eigen(Gw)$vectors[,3])

       # Plots of the FPC harmonics
X11()
par(mfrow=c(1,2))
plot(seq(0,120,30),psi_1[,1],type="l",ylim=c(-0.7,0.8),col="dark blue"    ,
               main=paste("Subj FPC",1:dim.space_b,", based on Gb, % variance:",round(Gbpst[1:dim.space_b],2)),ylab="",xlab="")
lines(seq(0,120,30),psi_1[,1],type="l",ylim=c(-1,1),lw=12,col="dark blue")
lines(seq(0,120,30),psi_1[,2],lw=6,col="blue")
X11()
plot(seq(0,120,30),psi_2[,1],type="l",ylim=c(-0.7,0.8),col="dark blue"    ,
               main=paste("Subj/visit FPC",1:dim.space_w,", based on Gw, % variance:",round(Gwpst[1:dim.space_w],2)),ylab="",xlab="")
lines(seq(0,120,30),psi_2[,1],type="l",ylim=c(-1,1),lw=12,col="dark blue")
lines(seq(0,120,30),psi_2[,2],lw=6,col="blue")
lines(seq(0,120,30),psi_2[,3],lw=2,col="light blue")

psi_subj                             <- psi_1
psi_subvis_w                         <- psi_2


       # Minimal smoothing of FPC vectors, to optain continuous FPC curves in the plots
       # (Necessary due to the small number of glucose measurements per woman)

psi.subj.smooth                      <- smooth.basis(c(0,30,60,90,120),psi_subj,fdParobj.m )
psi.subvis.smooth.w                  <- smooth.basis(c(0,30,60,90,120),psi_subvis_w,fdParobj.m )

       # Plots of the (minimally smoothed) FPC harmonics
X11()
par(mfrow=c(1,2))
plot(psi.subj.smooth)                                              # Empirical basis functions, subject level
plot(psi.subvis.smooth.w)                                          # Empirical basis functions, subj/visit level
```

```
       # Plot of mean curves + or - 2*SD of FPCs
X11()
par(mfrow=c(3,3))
evb                          <- eigen(Gb)$values/sum(eigen(Gb)$values[1:4])
evgw                         <- eigen(Gw)$values/sum(eigen(Gw)$values[1:5])
for(i in 1:dim.space_b){
plot(overallmean.eval.smooth$fd      ,col="grey", ylim=c(2.8,8.5),lw=6,ylab=paste("Glucose (mmol/l)"),xlab="Time (min)",
                             main=paste("Overall mean \n Subject-specific FPC",i))
points(6*seq(0:20)-6, eval.fd(6*seq(0:20)-6,
       smooth.basis(c(0,30,60,90,120),(overallmean+2*sqrt(eigen(Gb)$values[i])   *(psi_subj[,i])),fdParobj.m)$fd ), pch="+")
points(6*seq(0:20)-6, eval.fd(6*seq(0:20)-6,
       smooth.basis(c(0,30,60,90,120),(overallmean-2*sqrt(eigen(Gb)$values[i])   *(psi_subj[,i])),fdParobj.m)$fd ), pch="-")
}
plot.new()
for(i in 1:dim.space_w){
plot(visitspecificmean1.eval.smooth$fd      ,col="grey", ylim=c(2.8,8.5),lw=6,ylab=paste("Glucose (mmol/l)"), xlab="Time (min)",
                             main=paste("Mean, wks 14-16 \n Subj- and visit-specific FPC",i))
points(6*seq(0:20)-6, eval.fd(6*seq(0:20)-6,
       smooth.basis(c(0,30,60,90,120),(visitspecificmean1+2*sqrt(eigen(Gw)$values[i])   *(psi_subvis_w[,i])),fdParobj.m)$fd ), pch="+")
points(6*seq(0:20)-6, eval.fd(6*seq(0:20)-6,
       smooth.basis(c(0,30,60,90,120),(visitspecificmean1-2*sqrt(eigen(Gw)$values[i])   *(psi_subvis_w[,i])),fdParobj.m)$fd ), pch="-")
}

for(i in 1:dim.space_w){
plot(visitspecificmean3.eval.smooth$fd      ,col="grey", ylim=c(2.8,8.5),lw=6,ylab=paste("Glucose (mmol/l)"),xlab="Time (min)",
                             main=paste("Mean, wks 30-32 \n Subj- and visit-specific FPC",i))
points(6*seq(0:20)-6, eval.fd(6*seq(0:20)-6,
       smooth.basis(c(0,30,60,90,120),(visitspecificmean3+2*sqrt(eigen(Gw)$values[i])   *(psi_subvis_w[,i])),fdParobj.m)$fd ), pch="+")
points(6*seq(0:20)-6, eval.fd(6*seq(0:20)-6,
       smooth.basis(c(0,30,60,90,120),(visitspecificmean3-2*sqrt(eigen(Gw)$values[i])   *(psi_subvis_w[,i])),fdParobj.m)$fd ), pch="-")
}
```

```
          ### WINBUGS

dim.space_b                  <- 2   # Antall egenfunksjoner, level 1 subject-specific
dim.space_w                  <- 3   # Antall egenfunksjoner, level 2 subject/visit-specific

psi_subj                     <- psi_subj
psi_subvis                   <- psi_subvis_w

          # The matrices W_1 and W_2 contain centered data from visits 1 and 2, respectively..

W_1                          <- as.matrix(g1.demeaned) # dim 884,5
W_2                          <- as.matrix(g3.demeaned) # dim 884,5

          # Define the data, which contains the dimension of the level 1 space, dim.space_b, the dimension of the level 2 space, dim.space_w,
          # the level 1 and 2 eigenfunctions, psi_1 and psi_2, the data matrices for visit 1 and 2,
          # the number of subjects, N_subj, the maximum number of observations per subject, N_obs

data                         <- list("dim.space_b","dim.space_w","psi_subj","psi_subvis","W_1","W_2","N_subj","N_obs")

          # Define the program file (see below)

program.file.name            <- "M:/mfpca_n884_2fpcLevel1_3fpcLevel2.txt"       # See code below


          # Define the initial values

inits.W_1                    <- matrix(rep(NA,N_subj*N_obs),ncol=N_obs)
inits.W_1[is.na(W_1)]        <- mean(mean(W_1,na.rm=TRUE))

inits.W_2                    <- matrix(rep(NA,N_subj*N_obs),ncol=N_obs)
inits.W_2[is.na(W_2)]        <- mean(mean(W_2,na.rm=TRUE))

inits.ll_b                   <- rep(0.01,dim.space_b)
inits.ll_w                   <- rep(0.01,dim.space_w)

inits                        <-function(){list(xi=matrix(rep(0,N_subj*dim.space_b),ncol=dim.space_b),
                                    zi=array(rep(0,N_subj*dim.space_w*2),c(N_subj,dim.space_w,2)),
                                    taueps=0.01,ll_b=inits.ll_b,ll_w=inits.ll_w,W_1=inits.W_1,W_2=inits.W_2)}

          # Define the parameters to be monitored

parameters=list("lambda_b","xi[1:884,]","zi[1:884,,]")
#parameters=list("lambda_b","xi[1:11,]","zi[1:11,,]") (see comment on monitoring/convergence below)

library(R2WinBUGS) # May need to install it first: install.packages("R2WinBUGS")
```

```
        # Define the thinning, iteration and burn-in numbers for the MCMC simulation

set.seed(2708)              # choose a number
n.thin        <- 100        # this number is based on test-runs with close monitoring of a selected sub-sample of some of the parameters
n.iter        <- 105000     # chosen on basis of the thinning, burn-in
n.burnin      <- 5000       # convergence begins to stabilize around 2500, some structure in some curves until 3500, chooses 5000 to be sure.

ptm <- proc.time()

Bayes.fit     <- bugs(data, inits, parameters, model.file = program.file.name,
                      n.chains = 1, n.iter = n.iter, n.burnin = n.burnin,
                      n.thin = n.thin, debug = FALSE, DIC = FALSE, digits = 5,
                      codaPkg = FALSE,
                      bugs.directory = "D:/winbugs14/WinBUGS14/")

proc.time() - ptm

autocorr.plot(as.mcmc.list(Bayes.fit), lag.max=50, auto.layout = TRUE)    # only works if few parameters are monitored,
                                                                           # e.g. "lambda_b","xi[1:11,]","zi[1:11,,]"
print(Bayes.fit)
plot(Bayes.fit)
head(Bayes.fit)
attach.bugs(Bayes.fit)

scores.l1.subj              <- colMeans(xi)             # Subject-specific FPC scores
scores.l2.subj.v1           <- colMeans(zi)[,,1]        # Subject- and visit-specific FPC scores, visit 1
scores.l2.subj.v3           <- colMeans(zi)[,,2]        # Subject- and visit-specific FPC scores, visit 3

       # Correlation table

round(cor(cbind(eval.g1,eval.g3,spssdata.term.complete.ogtt$auc1,spssdata.term.complete.ogtt$auc3,
                                                    scores.l1.subj,scores.l2.subj.v1,scores.l2.subj.v3) ),2)

       # Save FPC scores

scores.mfpca                <- cbind(spssdata.term.complete.ogtt$id,scores.l1.subj,scores.l2.subj.v1,scores.l2.subj.v3)
write.table(scores.mfpca, file="M:/Art5longitudinalFDA/Bayes_FDA/R2WinBUGS/scores.mfpca.may2013.csv")

       # Read saved FPC scores from file
scores.mfpca                <- read.table("M:/Art5longitudinalFDA/Bayes_FDA/R2WinBUGS/scores.mfpca.may2013.csv")
```

```
### FIGURE 2

X11()   #       The 2*884 smoothed glucose curves
par(mar=c(1,1,1,1))
plot(g1.smooth,lty=1,col="black",lw=2,ylim=c(-2,12.5),xaxt='n', yaxt='n',ann=FALSE)
plot(g3.smooth,lty=1,col="black",lw=2,add=TRUE)
abline(h=0,col="grey",lw=5)

X11()   #       Overall mean
par(mar=c(1,1,1,1))
plot(overallmean.eval.smooth,lty=1,lw=5,col="black",ylim=c(-2,12.5),xaxt='n', yaxt='n',ann=FALSE)
abline(h=0,col="grey",lw=5)

X11()   #       eta
par(mar=c(1,1,1,1))
plot(visitspecificmean1.eval.smooth$fd-overallmean.eval.smooth$fd,lty=1,lw=5,col="black",ylim=c(-2,12.5),xaxt='n', yaxt='n',ann=FALSE)
plot(visitspecificmean3.eval.smooth$fd-overallmean.eval.smooth$fd,lty=1,lw=5,col="black",xaxt='n', yaxt='n',ann=FALSE,add=TRUE)
abline(h=0,col="grey",lw=5)


X11()   #       The estimated X-curves
par(mar=c(1,1,1,1))
plot(scores.mfpca[884,2]*psi.subj.smooth$fd[1]+scores.mfpca[884,3]*psi.subj.smooth$fd[2],
               lty=1,lw=2,col="black",ylim=c(-2,12.5),xaxt='n', yaxt='n',ann=FALSE)
for(i in 1:884){
plot(scores.mfpca[i,2]*psi.subj.smooth$fd[1]+scores.mfpca[i,3]*psi.subj.smooth$fd[2],lty=1,lw=2,col="black",xaxt='n',yaxt='n',ann=FALSE,add=TRUE)}
abline(h=0,col="grey",lw=5)

X11()   #       The estimated U-curves
par(mar=c(1,1,1,1))
plot(scores.mfpca[828,4]*psi.subvis.smooth.w$fd[1]+scores.mfpca[828,5]*psi.subvis.smooth.w$fd[2]+scores.mfpca[828,6]*psi.subvis.smooth.w$fd[3],
               lty=1,lw=2,col="black",ylim=c(-2,12.5),xaxt='n', yaxt='n',ann=FALSE)
plot(scores.mfpca[828,7]*psi.subvis.smooth.w$fd[1]+scores.mfpca[828,8]*psi.subvis.smooth.w$fd[2]+scores.mfpca[828,9]*psi.subvis.smooth.w$fd[3],
               lty=1,lw=2,col="black", xaxt='n', yaxt='n',ann=FALSE,add=TRUE)
for(i in 1:884){
plot(scores.mfpca[i,4]*psi.subvis.smooth.w$fd[1]+scores.mfpca[i,5]*psi.subvis.smooth.w$fd[2]+scores.mfpca[i,6]*psi.subvis.smooth.w$fd[3],
               lty=1,lw=2,col="black", xaxt='n', yaxt='n',ann=FALSE,add=TRUE)
plot(scores.mfpca[i,7]*psi.subvis.smooth.w$fd[1]+scores.mfpca[i,8]*psi.subvis.smooth.w$fd[2]+scores.mfpca[i,9]*psi.subvis.smooth.w$fd[3],
               lty=1,lw=2,col="black", xaxt='n', yaxt='n',ann=FALSE,add=TRUE)
}
abline(h=0,col="grey",lw=5)

X11()   #       Visit-specific means
par(mar=c(1,1,1,1))
plot(visitspecificmean1.eval.smooth,lty=1,lw=5,col="black",ylim=c(-2,12.5),xaxt='n', yaxt='n',ann=FALSE)
plot(visitspecificmean3.eval.smooth,lty=1,lw=5,col="black", xaxt='n', yaxt='n',ann=FALSE,add=TRUE)
abline(h=0,col="grey",lw=5)

X11()   #       B-splines-smoothed curves for woman no 828
par(mar=c(1,1,1,1))
plot(g1.smooth$fd[828],lty=1,lw=5,col="black",ylim=c(-2,12.5),xaxt='n', yaxt='n',ann=FALSE)
plot(g3.smooth$fd[828],lty=1,lw=5,col="black",add=TRUE)
```

```
abline(h=0,col="grey",lw=5)

X11()   #       Estimated X-curve for woman no 828
par(mar=c(1,1,1,1))
plot(scores.mfpca[828,2]*psi.subj.smooth$fd[1]+scores.mfpca[828,3]*psi.subj.smooth$fd[2],
                lty=1,lw=5,col="black",ylim=c(-2,12.5),xaxt='n', yaxt='n',ann=FALSE)
abline(h=0,col="grey",lw=5)

X11()   #       mu(t) + eta(t) + Xhat(t) for woman no 828
par(mar=c(1,1,1,1))
plot(visitspecificmean1.eval.smooth$fd+scores.mfpca[828,2]*psi.subj.smooth$fd[1]+scores.mfpca[828,3]*psi.subj.smooth$fd[2],
                lty=1,lw=5,col="black",ylim=c(-2,12.5),xaxt='n', yaxt='n',ann=FALSE)
plot(visitspecificmean3.eval.smooth$fd+scores.mfpca[828,2]*psi.subj.smooth$fd[1]+scores.mfpca[828,3]*psi.subj.smooth$fd[2],
                lty=1,lw=5,col="black", xaxt='n', yaxt='n',ann=FALSE,add=TRUE)
abline(h=0,col="grey",lw=5)

X11()   #       Estimated U-curves for woman no 828
par(mar=c(1,1,1,1))
plot(scores.mfpca[828,4]*psi.subvis.smooth.w$fd[1]+scores.mfpca[828,5]*psi.subvis.smooth.w$fd[2]+scores.mfpca[828,6]*psi.subvis.smooth.w$fd[3],
                lty=1,lw=5,col="black",ylim=c(-2,12.5),xaxt='n', yaxt='n',ann=FALSE)
plot(scores.mfpca[828,7]*psi.subvis.smooth.w$fd[1]+scores.mfpca[828,8]*psi.subvis.smooth.w$fd[2]+scores.mfpca[828,9]*psi.subvis.smooth.w$fd[3],
                lty=1,lw=5,col="black", xaxt='n', yaxt='n',ann=FALSE,add=TRUE)
abline(h=0,col="grey",lw=5)

X11()   #       mu(t) + eta(t) + Xhat(t) + Uhat(t) for woman no 828
par(mar=c(1,1,1,1))
plot(visitspecificmean1.eval.smooth$fd+scores.mfpca[828,2]*psi.subj.smooth$fd[1]+scores.mfpca[828,3]*psi.subj.smooth$fd[2]+

        scores.mfpca[828,4]*psi.subvis.smooth.w$fd[1]+scores.mfpca[828,5]*psi.subvis.smooth.w$fd[2]+scores.mfpca[828,6]*psi.subvis.smooth.w$fd[3],
                lty=1,lw=5,col="black",ylim=c(-2,12.5),xaxt='n', yaxt='n',ann=FALSE)
plot(visitspecificmean3.eval.smooth$fd+scores.mfpca[828,2]*psi.subj.smooth$fd[1]+scores.mfpca[828,3]*psi.subj.smooth$fd[2]+

        scores.mfpca[828,7]*psi.subvis.smooth.w$fd[1]+scores.mfpca[828,8]*psi.subvis.smooth.w$fd[2]+scores.mfpca[828,9]*psi.subvis.smooth.w$fd[3],
                lty=1,lw=5,col="black", xaxt='n', yaxt='n',ann=FALSE,add=TRUE)
abline(h=0,col="grey",lw=5)

X11()   #       B-splines-smoothed curves for woman no 828 and mu(t) + eta(t) + Xhat(t) + Uhat(t) for woman no 828 in the same plot
par(mar=c(1,1,1,1))
plot(g1.smooth$fd[828],lty=1,lw=5,col="black",ylim=c(-2,12.5),xaxt='n', yaxt='n',ann=FALSE)
plot(g3.smooth$fd[828],lty=1,lw=5,col="black",add=TRUE)
plot(visitspecificmean1.eval.smooth$fd+scores.mfpca[828,2]*psi.subj.smooth$fd[1]+scores.mfpca[828,3]*psi.subj.smooth$fd[2]+

        scores.mfpca[828,4]*psi.subvis.smooth.w$fd[1]+scores.mfpca[828,5]*psi.subvis.smooth.w$fd[2]+scores.mfpca[828,6]*psi.subvis.smooth.w$fd[3],
                lty=2,lw=5,col="black",xaxt='n', yaxt='n',ann=FALSE,add=TRUE)
plot(visitspecificmean3.eval.smooth$fd+scores.mfpca[828,2]*psi.subj.smooth$fd[1]+scores.mfpca[828,3]*psi.subj.smooth$fd[2]+

        scores.mfpca[828,7]*psi.subvis.smooth.w$fd[1]+scores.mfpca[828,8]*psi.subvis.smooth.w$fd[2]+scores.mfpca[828,9]*psi.subvis.smooth.w$fd[3],
                lty=2,lw=5,col="black", xaxt='n', yaxt='n',ann=FALSE,add=TRUE)
abline(h=0,col="grey",lw=5)
```

The program file, "M:/mfpca_n884_2fpcLevel1_3fpcLevel2.txt":

```
model
{#Start model
       for (i in 1:N_subj)
    {for (t in 1:N_obs)
        {W_1[i,t]~dnorm(m_1[i,t],taueps)
         W_2[i,t]~dnorm(m_2[i,t],taueps)

         m_1[i,t]<-X[i,t]+U_1[i,t]
         m_2[i,t]<-X[i,t]+U_2[i,t]

         X[i,t]       <-xi[i,1]*psi_subj[t,1]+xi[i,2]*psi_subj[t,2]

         U_1[i,t]     <-zi[i,1,1]*psi_subvis[t,1]+zi[i,2,1]*psi_subvis[t,2]+zi[i,3,1]*psi_subvis[t,3]

         U_2[i,t]     <-zi[i,1,2]*psi_subvis[t,1]+zi[i,2,2]*psi_subvis[t,2]+zi[i,3,2]*psi_subvis[t,3]
        }

     for (k in 1:dim.space_b)
        {xi[i,k]~dnorm(0,ll_b[k])}

     for (l in 1:dim.space_w)
        {zi[i,l,1]~dnorm(0,ll_w[l])
         zi[i,l,2]~dnorm(0,ll_w[l])}
        }#

for (k in 1:dim.space_b)
       {ll_b[k]~dgamma(1.0E-3,1.0E-3)
        lambda_b[k]<-1/ll_b[k]}

for (l in 1:dim.space_w)
       {ll_w[l]~dgamma(1.0E-3,1.0E-3)
        lambda_w[l]<-1/ll_w[l]}

taueps~dgamma(1.0E-3,1.0E-3)
sigma_sq_eps<-1/taueps
}#End model
```
